# Particulate Air Pollution, Ambulatory Heart Rate Variability, and Cardiac Arrhythmia in Retirement Community Residents with Coronary Artery Disease

**DOI:** 10.1289/ehp.1205914

**Published:** 2013-07-09

**Authors:** Scott M. Bartell, John Longhurst, Thomas Tjoa, Constantinos Sioutas, Ralph J. Delfino

**Affiliations:** 1 Program in Public Health, and; 2 Department of Statistics, University of California, Irvine, Irvine, California, USA; 3 Department of Epidemiology; 4 Susan Samueli Center for Integrative Medicine, and; 5 Cardiology Division, Department of Medicine, School of Medicine, University of California, Irvine, Irvine, California, USA; 6 Department of Civil and Environmental Engineering, Viterbi School of Engineering, University of Southern California, Los Angeles, California, USA

## Abstract

Background: Decreased heart rate variability (HRV) has been associated with future cardiac morbidity and mortality and is often used as a marker of altered cardiac autonomic balance in studies of health effects of airborne particulate matter. Fewer studies have evaluated associations between air pollutants and cardiac arrhythmia.

Objectives: We examined relationships between cardiac arrhythmias, HRV, and exposures to airborne particulate matter.

Methods: We measured HRV and arrhythmia with ambulatory electrocardiograms in a cohort panel study for up to 235 hr per participant among 50 nonsmokers with coronary artery disease who were ≥ 71 years of age and living in four retirement communities in the Los Angeles, California, Air Basin. Exposures included hourly outdoor gases, hourly traffic-related and secondary organic aerosol markers, and daily size-fractionated particle mass. We used repeated measures analyses, adjusting for actigraph-derived physical activity and heart rate, temperature, day of week, season, and community location.

Results: Ventricular tachycardia was significantly increased in association with increases in markers of traffic-related particles, secondary organic carbon, and ozone. Few consistent associations were observed for supraventricular tachycardia. Particulates were significantly associated with decreased ambulatory HRV only in the 20 participants using ACE (angiotensin I–converting enzyme) inhibitors.

Conclusions: Although these data support the hypothesis that particulate exposures may increase the risk of ventricular tachycardia for elderly people with coronary artery disease, HRV was not associated with exposure in most of our participants. These results are consistent with previous findings in this cohort for systemic inflammation, blood pressure, and ST segment depression.

Citation: Bartell SM, Longhurst J, Tjoa T, Sioutas C, Delfino RJ. 2013. Particulate air pollution, ambulatory heart rate variability, and cardiac arrhythmia in retirement community residents with coronary artery disease. Environ Health Perspect 121:1135–1141; http://dx.doi.org/10.1289/ehp.1205914

## Introduction

Decreased heart rate variability (HRV) has been associated with cardiac morbidity and mortality and is often used as a marker of autonomic dysfunction in the assessment of the impact of air pollution on cardiac autonomic control (Link and Dockery 2010). Associations also have been found between HRV and ischemia in coronary artery disease (CAD) patients who are monitored by ambulatory electrocardiograms (ECGs), including findings of asymptomatic ST segment depression indicative of cardiac ischemia (Vardas et al. 1996). Given that many patients with CAD already have decreased HRV, pollutant exposures that lead to further HRV decreases might be expected to precipitate adverse clinical events such as ventricular tachycardia and myocardial ischemia and infarction. Indeed, a number of recent air pollution studies have focused on CAD patients, although with mixed findings regarding associations between air pollutants and HRV (Barclay et al. 2009; Folino et al. 2009; Hampel et al. 2010; Schneider et al. 2010; Zanobetti et al. 2010). Nevertheless, in a recent review of the literature on particulate air pollution and cardiovascular outcomes, Brook et al. (2010) concluded that there was strong epidemiological evidence for an association between decreased HRV and short-term exposures to particulate matter (PM) air pollution in various populations, with the most consistent findings in older or clinically susceptible populations.

In contrast, Brook et al. (2010) also concluded that there was limited or weak epidemiological evidence for associations of cardiac arrhythmia with short-term exposures to PM. In another review, Link and Dockery (2010) suggested that there was evidence of an association of PM with cardiac arrhythmias, particularly ventricular arrhythmias, in patients with underlying cardiac disease. Studies published since those reviews had mixed results: He et al. (2011) reported a significant association between personal PM_2.5_ (PM with an aerodynamic diameter of ≤ 2.5 μm) exposure and premature ventricular contractions among 105 middle-aged participants using ambulatory ECGs, but only among participants without cardiovascular disease, and Mills et al. (2011) reported no “serious arrhythmias” or HRV effects in a randomized trial of dilute diesel exhaust exposure with 52 middle-aged participants. Little is known about possible mechanisms for PM effects on arrhythmia, although altered HRV, repolarization abnormalities, oxidative stress, and myocardial ischemia have been proposed as contributing factors (Link and Dockery 2010).

Between 2005 and 2007 we conducted a cohort panel study of acute cardiovascular outcomes with home-based ambient air pollution monitoring in the Los Angeles, California, Air Basin, where ambient air pollution is dominated by mobile sources. Our focus was on traffic-related ultrafine particles, which are particularly high in redox-active chemical components (Ayres et al. 2008; Verma et al. 2009) that are hypothesized to induce arrhythmias via lipid peroxidation, endothelial dysfunction, and other mechanisms involving oxidative stress (Griendling and FitzGerald 2003). In the present study, we used data from the same Los Angeles cohort to study the hypothesis that increased cardiac arrhythmia and decreased HRV are associated with exposure to PM.

## Materials and Methods

*Study population.* We made repeated ambulatory ECG measurements among 55 elderly nonsmokers with CAD who were recruited from four retirement communities in the Los Angeles Air Basin. Eligibility criteria included age of ≥ 65 years, history of CAD, being a nonsmoker, and having no exposure to environmental tobacco smoke. CAD diagnoses were obtained by questionnaire or cardiologist interview, and were confirmed with medical records review as described previously (Delfino et al. 2008). Study cardiologists and nurses clinically evaluated 105 potentially eligible participants in our mobile medicine clinic. Twenty-one participants were not eligible to participate in the overall study, 18 dropped out of the ambulatory ECG monitoring portion of the study, and 4 had insufficient ambulatory ECG monitoring time (≤ 3 days). Twelve participants who had pacemakers also were excluded because pacemakers can render arrhythmia and HRV data invalid, leaving 50 participants and 8,952 hr of data. We confirmed the presence and absence of pacemakers using baseline ECG and ambulatory ECG data, and used digital and paper diaries to monitor daily medication use.

This study was approved by the institutional review board of the University of California, Irvine, in 2003. All participants gave informed consent.

*Ambulatory ECG monitoring and outcomes.* Each participant wore an ambulatory ECG (Holter) monitors for two 5-day periods (Sunday through Friday) during 12 weeks of follow-up: one 5-day period between July and mid-October and one 5-day period between mid-October and February. We used the Burdick model 92513 compact digital Holter recorder and scanner/software system (Burdick Inc., Deerfield, WI), which is a seven-lead, three-channel Holter ECG that has a data acquisition speed of 200 Hz. Each day, the subject removed leads and bathed before a trained research assistant arrived at the subject’s home to download ECG data and setup the ECG for a new day. The Holter ECG signals (three channels) were read and analyzed using the Burdick Vision Premier Holter Analysis System, which includes algorithms for QRS labeling, arrhythmia detection, artifact identification, and data correction. A Holter technician from the Noninvasive Laboratory of the University of California, Irvine, Medical Center then screened the data for artifacts and outliers; indications of abnormalities, arrhythmias, ST segment changes and flagged ECG regions were reviewed by physicians.

Arrhythmia outcomes included supraventricular and ventricular arrhythmias in consecutive runs of ≥ 3 ectopic beats at a rate ≥ 120 beats per min (bpm) (Berger et al. 2006). The duration of arrhythmias and the number of foci in each hour were recorded. Numbers of arrhythmia events during 24-hr intervals corresponding to the gravimetric particle mass sampling times (afternoon to afternoon) also were recorded. The Holter output for each episode of supraventricular (atrial) tachycardia (SVT) and ventricular tachycardia (VT) was extracted. We defined SVT as paroxysmal supraventricular tachycardia (observed in *n* = 460 subject-hours with one or more foci), and VT as single focus ventricular tachycardia (*n* = 69 subject-hours) or multifocal ventricular tachycardia (*n* = 232 subject-hours). Atrial fibrillation (*n* = 2 subject-hours), atrial flutter (*n* = 2 subject-hours), atrioventricular block (*n* = 12 subject-hours), and accelerated idioventricular rhythm (*n* = 50 subject-hours) were observed too infrequently to analyze; we calculated subject-hours, including SVT or VT, without regard to these rare events.

We used Burdick Vision Series HRV software to analyze the QRS annotation file described above. All HRV measures were derived from 5-min epochs using acceptable normal-to-normal (N-N) intervals (Task Force of the European Society of Cardiology and the North American Society of Pacing and Electrophysiology 1996). Only beats classified as normal and not preceded by ectopic beats or prolonged R-R intervals were included in the N-N interval estimation. An interpolation algorithm using cubic splines was used to replace missing beats, including the removed ectopic beats and artifacts, premature ventricular contractions (PVCs), and other nonnormal R-R areas (Albrecht and Cohen 1988). N-N intervals < 300 and/or > 3,000 msec or with N-N ratios between < 0.8 or > 1.2 were rejected. Segments in which > 20% of R-R intervals required replacement were excluded from the analysis.

The HRV outcomes consisted of standard time domain variables (Task Force of the European Society of Cardiology and the North American Society of Pacing and Electrophysiology 1996) measured at the hourly level: standard deviation of normal R-R intervals (SDNN), root mean square of the successive differences between R-R intervals (r-MSSD), and logit percentage of adjacent normal R-R intervals that differed by > 50 msec (logit pNN50). HRV outcomes were computed both for 1- and 24-hr intervals spanning the pollutant sampling periods (described below). However, day/night differences in N-N intervals can contribute to a major fraction of the SDNN magnitude (Kleiger et al 2005). Therefore, for pollutants that were measured hourly, we also conducted analyses that were stratified according to daytime (0600–1900 hours) and nighttime (1900–0600 hours) periods.

The distributions of SDNN, r-MSSD, and pNN50 were moderately right skewed. After removing some extreme outliers, we used three times the median of the absolute deviations of each SDNN or r-MSSD measurement from their median as a cut point for further removal of outliers (Zanobetti et al. 2010). This resulted in a loss of approximately 11% of hourly SDNN records and 13% of hourly r-MSSD records. The logit transformation was used for pNN50 with 2.27% of the hourly pNN50 records excluded because of pNN50 = 0.

*Environmental exposures.* We monitored outdoor air pollutants using a specially equipped trailer parked at each retirement community. Hourly particle exposures for the analysis of both hourly and 24-hr HRV outcomes included total particle number concentrations (condensation particle counter model 3785; TSI Inc., Shoreview, MN), PM_2.5_ black carbon (BC) (Aethalometer; Magee Scientific, Berkeley, CA), PM_2.5_ organic and elemental carbon (OC-EC Analyzer model 3F; Sunset Laboratory Inc., Tigard, OR) (Arhami et al. 2006), and PM_2.5_ mass using a beta-attenuation mass monitor (model 1020; Met One Instruments Inc., Grants Pass, OR). The particle counter detects particle sizes ranging from 5 nm to > 3,000 nm. We measured hourly pollutant gases including ozone (O_3_) and markers of fossil fuel combustion, namely carbon monoxide (CO) and nitrogen oxides (NO_x_), using federal reference methods (U.S. Environmental Protection Agency 2013). BC and elemental carbon (EC) are similar, but not identical, measurements of carbon linked to fossil fuel combustion.

We used the Sioutas™ Personal Cascade Impactor Sampler (SKC Inc., Eighty Four, PA) to measure daily size-fractionated PM mass concentrations (Misra et al. 2002). The analysis included particles 0–0.25 μm in diameter (PM_0.25_), accumulation mode particles 0.25–2.5 μm in diameter (PM_0.25–2.5_), and coarse mode particles 2.5–10 μm in diameter (PM_2.5–10_). PM_0.25_ is considered “quasi-ultrafine” because the traditional cut point for the ultrafine mode is around 0.1–0.2 μm. Meteorological data were also collected in the air sampling trailer.

We estimated the mass of total organic carbon (OC) attributed to secondary OC (a surrogate of photochemically derived OC) and the mass of OC attributed to primary OC (attributable to primary combustion sources, mostly traffic in the study regions). These estimates were based on the EC tracer method and are described elsewhere (Polidori et al. 2007).

*Personal accelerometer data.* Given the inverse relationship between physical activity and vagal influences on the heart during high levels of activity (Grossman and Taylor 2007; Grossman et al. 2004), it is possible that associations between air pollution and ambulatory HRV could be confounded or modified by different activity levels, especially respiratory sinus arrhythmia as reflected by r-MSSD or pNN50. As such, we electronically monitored physical activity continuously for each 24-hr period with a personal motion logger (Mini-motionlogger; Ambulatory Monitoring Inc., Ardsley, NY), a piezoelectric accelerometer that recorded objective measurements of movement intensity using the unit’s high sensitivity proportional integrating measure mode. We discarded accelerometer data at the beginning and end of each daily sampling period to avoid movement artifacts from equipment removal and setup. Valid accelerometer data were available for 92.8% of all hourly ECG records. We normalized 1-min data by subject-specific *z*-scores, and then determined average movement intensity for hourly periods corresponding to the hourly periods for the HRV and arrhythmia data.

*Statistical analysis.* Statistical analyses were conducted using SAS software, versions 9.2 and 9.3 (SAS Institute Inc., Cary, NC). Regression models were used to estimate associations between HRV and arrhythmia outcomes with interquartile range (IQR) increases in individual pollutants adjusted for potential confounding variables. Generalized estimating equations (implemented in PROC GENMOD) were used to account for within-subject correlations induced by the repeated measures design. A Gaussian variance structure was assumed for continuous HRV outcomes (SDNN, r-MSSD, and logit pNN50). For arrhythmia outcomes (VT and SVT), a quasi-Poisson variance was assumed for daily counts, and a quasi-binomial variance was assumed for the absence or presence of VT or SVT events during hourly intervals. Autoregressive correlation was assumed for residual errors from the same individual. For analyses of daily VT counts, we excluded data from one participant who had > 19 VT events/day during the warm season phase because they were highly influential (Cook’s D > 0.3). In addition, we excluded VT data from eight other participants who had values > 3 SD above the mean.

We estimated odds ratios (ORs) for the occurrence of one or more arrhythmia events during hourly intervals in association with pollutant concentrations during the hour before the outcome measurement, and with average pollutant concentrations during the 4 hr, 8 hr, 24 hr, 3 days, and 5 days before the outcome. We estimated daily rate ratios associated with size-fractionated PM exposures (PM_0.25_, PM_0.25–2.5_, and PM_2.5–10_) averaged during the current 24-hr interval (lag 0) and the previous 24-hr interval (lag 1; 25- to 48-hr average), and averaged over 2 days (during the current and previous 24 hr). Longer average exposures or lags > 1 day could not be assessed for PM fractions because PM filters were collected only during the 5 days of Holter monitoring. Both the risk of VT (e.g., Lampert et al. 1994) and air pollutant exposures (Polidori et al. 2007) are well known to differ between daytime and nighttime hours, leading us to investigate modification of pollutant associations with VT by daytime and nighttime hours as an *a priori* hypothesis. Thus, we fit additional models stratified by this variable and conducted formal hypothesis tests using Cochran’s Q statistic (Cochran 1954) to assess the significance of differences in daytime versus nighttime associations.

In secondary analyses, we used interaction terms to test for effect modification by sex, medication use, and self-reported comorbidities. For VT, we also repeated the analyses excluding daytime hours in which participants reported they were away from home.

All effect estimates were adjusted for hourly or daily average actigraph-derived physical activity and heart rate, temperature averaged over the exposure time period, day of week, 5-day study period (July to mid-October or mid-October through February), and retirement community. Exposures were decomposed using the Sheppard et al. (2005) mean-centering approach to implicitly adjust for potential confounding and mismeasurement of exposure by study phase and retirement community (see Supplemental Material, pp. 2–3). We also investigated hour of day as a possible confounder, but it was omitted from final models because of a lack of evidence of any confounding (i.e., there was little change in model coefficients). We assessed statistical significance using a 5% type I error rate per test, and 95% confidence intervals (CIs) are reported for point estimates. Because of the relatively large number of tests conducted (multiple pollutants, multiple outcomes, and multiple lag times), the percentage of tests yielding significant associations is reported for major categories of comparisons.

## Results

Nearly half of the study participants had had a previous myocardial infarction; the remainder qualified for enrollment based on coronary artery bypass graft or angioplasty, a positive angiogram or stress test, or clinical diagnosis of CAD (Table 1). In addition, most (68%) had hypertension, and nearly all (76%) had hypercholesterolemia. Most participants were male (62%) and overweight [mean body mass index (BMI) was 27; 20% were obese]. Cardiac medications were common, the majority taking β-adrenergic blocking agents (beta blockers) or hypocholesterolemic agents such as statins. Although the four retirement communities were nonsmoking facilities and none of the participants were current or recent smokers (within 12 months), 44% were former smokers.

**Table 1:** Characteristics of participants (*n* = 50).

Characteristic	Mean ± SD or *n* (%)
Age (years)	83.3 ± 5.95
BMI (kg/m^2^)	27.0 ± 3.63
Obese (BMI > 30)	10 (20)
Sex
Male	31 (62)
Female	19 (38)
Cardiovascular history
Confirmation of CAD^*a*^	
Myocardial infarction	23 (46)
Coronary artery bypass graft or angioplasty	16 (32)
Positive angiogram or stress test	8 (16)
Clinical diagnosis of coronary microvascular disease	3 (6)
Current angina pectoris	14 (28)
Cardiac arrhythmia	10 (20)
Congestive heart failure	10 (20)
Hypertension	34 (68)
Hypercholesterolemia	38 (76)
Other medical history
Type 2 diabetes	7 (14)
COPD or asthma	5 (10)
Stroke or transient ischemic attack	8 (16)
Medications
β-Adrenergic blocking agents	30 (60)
Antiarrhythmic drugs	4 (8)
Digoxin	3 (6)
ACE inhibitors	20 (40)
HMG-CoA reductase inhibitors (statins)	27 (54)
Platelet aggregation inhibitors or Coumadin	17 (34)
Calcium channel blockers	17 (34)
At least one beta-blocker or calcium channel blocker medication^*b*^	37 (74)
Smoking history
Never-smoker	28 (56)
Former smoker^*c*^	22 (44)
Abbreviations: ACE, angiotensin I–converting enzyme; COPD, chronic obstructive pulmonary disease; HMG-CoA, 3-hydroxy-3-methylglutaryl coenzyme A. ^***a***^Each category is hierarchical (excludes being in above diagnostic category). ^***b***^A beta-blocker, a calcium channel blocker, or an antiarrhythmic drug having beta-blocker and/or calcium channel antagonistic effects. ^***c***^No smoking within 12 months.	

Summary statistics for the outcome variables are shown in Table 2. pNN50 was similar for day and night, but SDNN and r-MSSD were higher for daytime hours than nighttime hours.

**Table 2:** Distribution of outcome variables.

Outcome	Mean ± SD	Median	Minimum	Maximum
VT^*a*^
No. during 24 hr	1.73 ± 11.14	0	0	158
SVT^*b*^
No. during 24 hr	1.32 ± 3.46	0	0	36
SDNN
Day and night^*c*^	72.91 ± 34.98	66	3	159
Daytime	74.26 ± 34.19	68	6	159
Nighttime	69.92 ± 36.50	62	3	159
r-MSSD
Day and night	49.25 ± 45.67	29	0.06	183
Daytime	50.32 ± 46.71	29	0.06	183
Nighttime	46.88 ± 43.18	29	0.11	183
pNN50
Day and night	22.4 ± 28.7	5.76	0	97.3
Daytime	22.5 ± 28.4	5.85	0	91.5
Nighttime	22.4 ± 28.9	5.65	0	97.3
^***a***^Based on VT events observed in 301 hourly periods among 18 participants. ^***b***^Based on SVT events observed in 460 hourly periods among 39 participants; 13 of those participants also experienced VT events. ^***c***^Daytime is 0600–1900 hours; nighttime is 1900–0600 hours.				

Pollutant measurements are described in Table 3. Because two or three participants in the same retirement community were simultaneously monitored on each day, the number of 24-hr pollutant measurements (*n* = 235) is < 500 measurements, which would have resulted from measuring each participant’s personal exposure. Regression effect estimates were standardized to the IQR of each pollutant. The traffic-related air pollutants (primary OC, NO_x_, CO) were highly positively correlated with each other, with pairwise Spearman correlations ≥ 0.75, whereas O_3_ was negatively correlated and secondary OC was not correlated with these primary combustion-related pollutants (Supplemental Material, Table S1). Given the strong correlation among primary pollutants, our regression models test one pollutant at a time.

**Table 3:** Distribution of outdoor air pollution variables.

Exposure (24-hr averages)^*a*^	*n*	*n* missing^*a*^	Mean ± SD	IQR	Minimum	Maximum
Continuously measured PM
PM_2.5_ (μg/m^3^)^*b*^	235	0	21.1 ± 11.4	16.1	2.3	77.4
Particle number (no./cm^3^)	184	51	12,818 ± 5,889	6,351	2,019	30,180
BC (μg/m^3^)	235	0	1.7 ± 0.8	1.0	0.3	4.5
EC (μg/m^3^)	198	37	1.52 ± 0.6	0.9	0.3	3.3
OC (μg/m^3^)	188	47	7.8 ± 3.7	5.2	2.5	18.7
Primary OC (μg/m^3^)	157	78	5.3 ± 2.9	4.4	41.4	12.6
Secondary OC (μg/m^3^)	157	78	2.9 ± 1.5	2.1	0.3	7.7
Size-fractionated PM (μg/m^3^)^*b*^
PM_0.25_	217	18	9.8 ± 4.1	7.0	2.5	30.1
PM_0.25–2.5_	226	9	11.4 ± 9.4	10.6	1.0	66.8
PM_2.5–10_	217	18	9.4 ± 5.0	5.5	0.3	24.6
Criteria pollutant gases
NO_x_ (ppb)	235	0	46.6 ± 31.4	42.3	3.2	183.7
CO (ppm)	224	11	0.53 ± 0.30	0.42	0.01	1.68
O_3_ (ppb)	232	3	27.1 ± 11.5	17.4	3.8	60.7
^***a***^The continuously measured PM variables were averaged hourly and are presented as 24-hr averages; there were fewer missing observations for BC, PM_2.5_, and the gases because two samplers were operated in parallel at all times; there was more missing primary and secondary OC than total OC because of missing predictor data used to estimate these two OC fractions, including EC. ^***b***^PM_2.5_ mass was measured hourly with a pair of beta-attenuation mass monitors, whereas the size-fractionated PM mass was measured daily with a personal cascade impactor sampler, which was missing more data.						

Associations between daily count of VT events and outdoor air pollutants, adjusted for confounding variables, are shown in Table 4. An IQR increase in 24-hr average PM_2.5_ was associated with a 50% increase in the daily rate of VT events (95% CI: 2, 20%). The estimated effect of 3-day average PM_2.5_ on daily VT was nearly identical, although not statistically significant. Analysis of PM_2.5_ constituents indicated statistically significant associations between daily VT and average exposures during the previous 24-hr for BC, EC, total OC, and primary OC. Average total OC during the previous 24-hr was strongly associated with daily VT [relative risk (RR) = 3.06; 95% CI: 1.82, 5.17 per IQR increase], with the strongest constituent-specific association being with primary OC and a relatively weak association with secondary OC. Ozone also was strongly associated with daily VT, particularly the 3-day average (RR = 2.95; 95% CI: 1.29, 6.74). Associations between size fractionated PM and daily VT varied by averaging time, ranging from a 0% to 31% increase for PM_0.25_ and PM_0.25–2.5_, and from a 13% decrease to 20% increase for PM_2.5–10_. When we included the outlying subject with > 19 events/day, RRs for VT events were mostly larger than reported here, often > 5 with wide CIs (data not shown).

**Table 4:** Associations^*a*^ of ventricular tachycardia with outdoor air pollutants, per IQR.

Exposure and averaging time	Daily RR (95% CI)	Hourly daytime^*b*^ OR (95% CI)	Hourly nighttime^*b*^ OR (95% CI)	*p*-Value^*c*^
PM_2.5_
1 hr	—	1.06 (0.83, 1.35)	0.94 (0.60, 1.47)	0.645
4 hr	—	1.21 (0.95, 1.54)	0.94 (0.56, 1.58)	0.391
8 hr	—	1.17 (0.95, 1.44)	0.86 (0.51, 1.45)	0.284
24 hr	1.50 (1.02, 2.20)**	1.23 (0.99, 1.52)*	0.85 (0.64, 1.12)	0.039**
3 day	1.51 (0.85, 2.70)	1.26 (0.99, 1.61)*	1.19 (0.84, 1.67)	0.778
5 day	1.16 (0.59, 2.29)	1.30 (0.78, 2.17)	2.15 (0.90, 5.11)*	0.328
Particle number
1 hr	—	1.06 (0.86, 1.30)	0.77 (0.59, 1.01)*	0.071*
4 hr	—	1.05 (0.84, 1.31)	0.90 (0.62, 1.30)	0.491
8 hr	—	0.90 (0.64, 1.26)	1.09 (0.70, 1.70)	0.506
24 hr	0.70 (0.41, 1.20)	0.90 (0.55, 1.46)	0.64 (0.31, 1.31)	0.442
3 day	0.42 (0.09, 1.94)	1.16 (0.41, 3.26)	0.70 (0.26, 1.92)	0.497
5 day	0.20 (0.02, 1.67)	2.43 (0.55, 10.7)	0.88 (0.10, 7.89)	0.453
BC
1 hr	—	1.07 (0.94, 1.22)	0.84 (0.53, 1.34)	0.327
4 hr	—	1.07 (0.91, 1.25)	0.75 (0.44, 1.29)	0.215
8 hr	—	1.23 (1.06, 1.44)^#^	0.69 (0.46, 1.03)*	0.008^#^
24 hr	1.40 (1.06, 1.84)**	1.30 (0.98, 1.72)*	0.74 (0.54, 1.00)*	0.008^#^
3 day	1.59 (0.77, 3.30)	1.88 (1.30, 2.73)^#^	1.38 (0.78, 2.42)	0.365
5 day	1.36 (0.64, 2.91)	2.68 (1.32, 5.42)^#^	2.63 (0.87, 8.00)*	0.978
EC
1 hr	—	1.01 (0.92, 1.11)	0.79 (0.56, 1.11)	0.181
4 hr	—	0.96 (0.85, 1.08)	0.80 (0.47, 1.35)	0.507
8 hr	—	1.09 (0.92, 1.30)	0.63 (0.38, 1.06)*	0.049**
24 hr	1.55 (1.09, 2.21)**	1.24 (0.95, 1.60)	0.69 (0.50, 0.96)**	0.006^#^
3 day	1.46 (0.65, 3.30)	2.10 (1.08, 4.09)**	1.09 (0.54, 2.22)	0.187
5 day	1.90 (0.82, 4.37)	3.07 (0.90, 10.5)*	1.57 (0.69, 3.58)	0.375
OC	
1 hr	—	1.24 (0.78, 1.97)	0.91 (0.49, 1.68)	0.431
4 hr	—	2.13 (1.31, 3.49)^#^	0.84 (0.38, 1.86)	0.051*
8 hr	—	2.58 (1.47, 4.52)^#^	0.65 (0.23, 1.81)	0.021**
24 hr	3.06 (1.82, 5.17)^#^	1.81 (1.08, 3.06)**	0.57 (0.19, 1.69)	0.060*
3 day	2.13 (0.60, 7.51)	2.76 (0.40, 19.02)	1.73 (0.65, 4.65)	0.673
5 day	5.95 (0.79, 44.8)*	4.22 (0.25, 71.7)	3.13 (0.25, 38.6)	0.877
Primary OC
1 hr	—	0.85 (0.65, 1.13)	0.89 (0.54, 1.46)	0.888
4 hr	—	0.93 (0.62, 1.39)	0.91 (0.44, 1.88)	0.966
8 hr	—	1.07 (0.54, 2.09)	0.64 (0.30, 1.37)	0.326
24 hr	2.64 (1.13, 6.21)**	1.90 (0.64, 5.61)	0.99 (0.34, 2.86)	0.400
3 day	2.16 (0.69, 6.77)	2.07 (0.57, 7.55)	0.78 (0.31, 1.98)	0.231
5 day	3.15 (0.30, 33.3)	11.2 (0.49, 258)	0.62 (0.07, 5.51)	0.137
Secondary OC
1 hr	—	1.18 (0.90, 1.54)	1.12 (0.76, 1.66)	0.833
4 hr	—	1.68 (1.21, 2.33)^#^	1.24 (0.76, 2.02)	0.307
8 hr	—	1.86 (1.06, 3.26)**	1.31 (0.90, 1.90)	0.301
24 hr	1.43 (0.93, 2.18)*	1.18 (0.69, 2.02)	1.45 (0.70, 3.00)	0.653
3 day	1.28 (0.47, 3.47)	0.68 (0.20, 2.30)	2.30 (0.41, 13.0)	0.261
5 day	2.27 (0.79, 6.58)	0.97 (0.22, 4.15)	1.68 (0.54, 5.23)	0.558
PM_0.25_
Lag 0 (24 hr)	1.04 (0.67, 1.60)	—	—
Lag 1 (25–48 hr)	1.20 (0.97, 1.47)*	—	—
2 day	1.29 (0.73, 2.29)	—	—
PM_0.25–2.5_
Lag 0 (24 hr)	1.31 (1.05, 1.64)**	—	—
Lag 1 (25–48 hr)	1.00 (0.78, 1.28)	—	—
2 day	1.30 (0.96, 1.77)*	—	—
PM_2.5–10_
Lag 0 (24 hr)	1.20 (0.90, 1.59)	—	—
Lag 1 (25–48 hr)	0.87 (0.71, 1.06)	—	—
2 day	0.97 (0.66, 1.44)	—	—
O_3_
1 hr	—	1.33 (1.09, 1.62)^#^	0.98 (0.66, 1.47)	0.184
4 hr	—	1.37 (1.06, 1.77)**	0.99 (0.64, 1.53)	0.213
8 hr	—	1.37 (0.98, 1.91)*	1.13 (0.74, 1.70)	0.472
24 hr	1.60 (1.12, 2.30)**	0.66 (0.35, 1.25)	2.68 (1.32, 5.46)^#^	0.004^#^
3 day	2.95 (1.29, 6.74)**	0.30 (0.08, 1.14)*	1.43 (0.45, 4.57)	0.082*
5 day	0.93 (0.10, 8.16)	0.05 (0.01, 0.53)**	2.05 (0.42, 9.86)	0.010**
NO_x_
1 hr	—	0.98 (0.75, 1.29)	1.13 (0.72, 1.78)	0.585
4 hr	—	0.99 (0.77, 1.27)	0.92 (0.55, 1.54)	0.800
8 hr	—	1.15 (0.90, 1.47)	0.67 (0.35, 1.27)	0.120
24 hr	1.37 (0.99, 1.88)*	1.49 (0.85, 2.62)	0.54 (0.28, 1.03)*	0.021**
3 day	1.80 (0.45, 7.12)	2.90 (0.87, 9.68)*	1.00 (0.30, 3.30)	0.219
5 day	1.70 (0.54, 5.40)	7.98 (1.48, 43.0)**	1.34 (0.14, 13.1)	0.217
CO
1 hr	—	1.03 (0.82, 1.30)	0.96 (0.66, 1.40)	0.730
4 hr	—	1.06 (0.84, 1.34)	0.79 (0.55, 1.15)	0.192
8 hr	—	1.11 (0.88, 1.39)	0.68 (0.47, 0.98)**	0.025**
24 hr	1.18 (0.84, 1.65)	0.84 (0.45, 1.56)	0.53 (0.32, 0.89)**	0.276
3 day	2.20 (0.82, 5.94)	1.03 (0.20, 5.35)	1.04 (0.30, 3.63)	0.994
5 day	0.84 (0.23, 3.03)	1.12 (0.14, 8.66)	1.06 (0.10, 11.0)	0.973
^***a***^All models were adjusted for daily average actigraph-derived physical activity and heart rate, temperature of the same lag average, day of week, seasonal study phase (mean centered exposure), and community group (mean centered exposure), using generalized estimating equations; daily RRs used Poisson log-link models with daily VT counts as the outcome, and hourly ORs used binomial logit-link models with hourly absence/presence of any VT as the outcome. ^***b***^Daytime is 0600–1900 hours; nighttime is 1900–0600 hours. ^***c***^*p*-Values are for heterogeneity of OR by day/night. *p < 0.1. **p < 0.05. ^#^*p* < 0.01.				

Table 4 also shows ORs for hourly daytime and nighttime models using occurrence of one or more VT events per hour as the outcome variable. In these models daytime VT risk was positively associated with IQR increases in PM_2.5_ concentrations for all averaging times, but none of the associations were statistically significant. Nighttime VT risk actually decreased with IQR increases in PM_2.5_ concentrations at averaging times shorter than 3 days, but again these associations were not statistically significant. Among PM constituents, BC and OC had the strongest associations with VT during the daytime and were significantly elevated across a variety of averaging times. NO_x_, a marker of primary combustion products, was positively associated with daytime VT for averaging times greater than 4 hr, but only the 5-day averaging time was statistically significant. In contrast, hourly nighttime VT was often negatively associated with PM constituents, to the extent that several formal tests of daytime–nighttime heterogeneity were statistically significant for 8- and 24-hr average exposures.

A mixture of daytime and nighttime positive associations were found for VT and O_3_. Daytime associations were positive for 1-hr, 4-hr, and 8-hr but negative for 24-hr, 3-day, and 5-day. In contrast, nighttime associations were nearly null at 1-hr and 4-hr and positive at 8-hr, 3-day, and 5-day. The inverse associations for 24-hr, 3-day, and 5-day O_3_ in daytime were unexpected.

In secondary analyses we tested other potential subject-specific effect measure modifiers but found no consistent differences in associations of VT with air pollutants according to sex, diabetes, obesity (BMI ≥ 30), and medication classes added to the model individually [antiarrhythmic medications, beta-blockers, digitalis and other inotropic agents, statins, and angiotensin I–converting enzyme (ACE) inhibitors) (data not shown)]. Although our panel study design was well powered to investigate associations with pollutants and interaction effects with stress and other time-varying characteristics, with only 50 participants it had little power to detect effect modification by sex, comorbidities, medications, and other time-invariant characteristics. We also repeated the VT analyses excluding daytime hours during which the participants reported being away from home (9% of daytime hours); results were very similar to those in Table 4 (data not shown).

Few significant associations were found between SVT and PM variables in either the daily or hourly models: None of 72 tests were significant at the 1% level, and only two tests (2.8%) were significant at the 5% level (Supplemental Material, Table S2). Notably, these two “significant” associations occurred at a rate near the expected 5% false positive rate for these tests under a null hypothesis of no associations. Among 27 tests for gaseous pollutants, there were four statistically significant decreases in SVT (for 3-day O_3_, 4-hr NO_x_, 1-hr CO, and 4-hr CO) and the only statistically significant increases in SVT were for IQR increases in 4-hr (RR = 1.19; 95% CI: 1.02, 1.39) and 8-hr ozone (RR = 1.25; 95% CI: 1.00, 1.56).

Few significant associations were estimated for PM variables and the three hourly HRV outcomes (SDNN, r-MSSD, and logit pNN50): None of 126 tests were significant at the 1% level, and only 6 tests (4.8%) were significant at the 5% level (see Supplemental Material, Table S3). In contrast, the 54 tests for outdoor gaseous pollutants (O_3_, NO_x_, and CO) with hourly HRV yielded 3 significant associations (5.6%) at the 1% level, and 9 significant associations (16.7%) at the 5% level, primarily showing unexpected increases in HRV associated with IQR increases in NO_x_ and CO exposure at 4- to 8-hr lags. Similarly, daily lag 0, 1, and 2-day average size-fractionated PM mass concentrations also were not significantly associated with 24-hr SDNN (data not shown).

**Figure 1:**
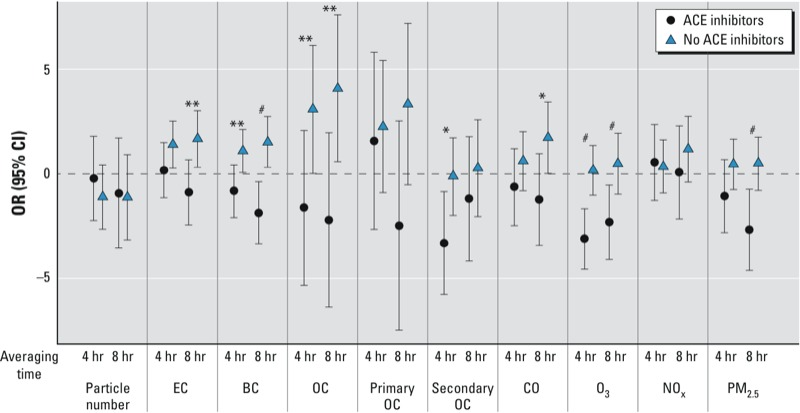
Associations of SDNN with outdoor air pollutants: effect modification by ACE inhibitor use. *p < 0.1, **p < 0.05, ^#^*p* < 0.01, compared with no effect modification by ACE inhibitor use.

We tested potential effect measure modification of air pollutant effects on 4-hr, 8-hr, and 1-day SDNN by medication use and found no significant differences except for ACE inhibitors (Figure 1). Eight-hour PM_2.5_, 8-hr BC, 4-hr secondary OC, and 4-hr and 8-hr ozone were significantly and inversely associated with hourly SDNN only among the 20 participants taking ACE inhibitors; in contrast, 4-hr and 8-hr EC, 8-hr BC, and 8-hr OC were significantly and positively associated with hourly SDNN among the participants not taking ACE inhibitors (*p*-values not shown).

## Discussion

Prior to 2005, few studies of HRV and air pollution monitored participants using ambulatory ECG. Instead, most studies recorded short ECG strips at rest during in-clinic visits to control for effects of physical activity on respiratory sinus arrhythmia (Delfino et al. 2005). Here, we opted for activity-adjusted ambulatory ECG collected during two 5-day intervals for each participant along with detailed home air pollutant monitoring, which allowed estimation of PM and PM constituent effects on both HRV and cardiac arrhythmias.

Apart from the participants taking ACE inhibitors, these data do not show convincing associations between outdoor pollutants and HRV or SVT after adjusting for likely confounding variables. We believe that our community-based exposure measurements are more accurate and our confounding controls are as good or better than those used in previous investigations, which often relied on ambient pollutant monitoring at central sites and lacked actigraph physical activity data. We recognize, however, that outdoor air pollutant concentrations measured at home are only a surrogate for personal exposure because they do not capture air pollutant exposures during time spent away from the home or indoor pollutant sources. VT associations with pollutants were similar when diary-reported hours away from home were excluded, suggesting that time spent away from home is not a confounder in this setting.

Barclay et al. (2009) suggested that the use of beta-blockers and other cardiac medications, such as digitalis, which is vagotonic, may mitigate adverse effects of PM on HRV. Consistent with this possibility, de Hartog et al. (2009) observed associations between PM and reduced SDNN only in CAD patients not taking beta-blockers. However, we did not find any evidence that beta-blockers or other cardiac medications modified associations between air pollutants and HRV, with the exception of ACE inhibitors.

We observed evidence of negative associations between air pollutants and SDNN among participants using ACE inhibitors, in contrast with null or positive associations with the same pollutants among non-users. A similar finding was reported in a recent ambulatory ECG study of 1,607 participants 50–72 years of age, in which 10-year average PM_10_ was associated with decreased HRV only among the 97 participants taking ACE inhibitors (Adam et al. 2012). These findings may be related to the well-known side effect of ACE inhibitor–induced cough. Several mechanisms for this side effect have been proposed, including the accumulation in the airways of the protussive mediators bradykinin and substance P, which are degraded by ACE, and/or activation of bradykinin receptors on pulmonary rapidly adapting vagal afferents by ACE inhibitors (Dicpinigaitis 2006; Hargreaves et al. 1992). Thus the apparent reduction in SDNN in response to air pollutant exposures among participants using ACE inhibitors might be explained by the stimulation of pulmonary vagal afferents by bradykinin, resulting in increased parasympathetic tone and decreased HRV.

We estimated strong associations between PM and VT in our participants. One study of patients with implanted cardiac defibrillators (ICDs) reported no association of VT with ambient air pollution (Metzger et al. 2007), whereas two other studies did (Dockery et al. 2005; Ljungman et al. 2008). Our participants (mean age: 83 years) were older than those in previous studies, and only one had an ICD (this subject had no VT events). Similarly, Folino et al. (2009) reported strong associations between PM and VT, but not HRV, in CAD patients in their Holter study. Another Holter study of CAD patients reported associations between PM exposures and VT, but did not report any findings on HRV (Berger et al. 2006). We previously reported that other clinically important outcomes were associated with PM in our study participants, including blood pressure, ischemic ST segment depression, and markers of systemic inflammation and platelet activation that might contribute to PM-related cardiac electrical effects (Delfino et al. 2009, 2010a, 2010b, 2011).

Evidence of day and night effect modification of associations between VT and many pollutants might result from differences in indoor infiltration conditions and air pollutant composition between daytime and nighttime. For example, personal exposure to O_3_ might differ substantially between the day and night because of the use of air conditioning and reduced time outdoors during hotter daytime hours and greater exposure to outdoor air during cooler nighttime hours. This might explain the unexpected inverse association between VT and outdoor O_3_ during the daytime. It might also reflect differences in cardiac response to air pollutants between waking and sleeping hours, although we do not know of any specific mechanism to explain this.

## Conclusions

Our findings support the hypothesis that particulate air pollutant exposure increases the risk of VT (consecutive runs of ≥ 3 ectopic beats at a rate ≥ 120 bpm) in a population of elderly persons with CAD. An increased rate of arrhythmia at higher PM concentrations has direct medical relevance and strong support in the present study. However, decreased HRV was not associated with air pollutants except in the subset of participants taking ACE inhibitors (*n* = 20). Future panel studies on cardiac effects of PM exposure might benefit from assessment of other mechanisms in addition to HRV.

## Supplemental Material

(365 KB) PDF
