# Supplementary material for: Particulate Air Pollution, Ambulatory Heart Rate Variability, and Cardiac Arrhythmia in Retirement Community Residents with Coronary Artery Disease
Source: Environ Health Perspect. 2013 Jul 9;121(10):1135–41. doi: 10.1289/ehp.1205914 (PMC3801451; doi:10.1289/ehp.1205914)
Supplement: (365 KB) PDF [file ehp.1205914.s001.pdf]

Supplemental Material

Particulate Air Pollution, Ambulatory Heart Rate Variability, and Cardiac Arrhythmia in Retirement Community Residents with Coronary Artery Disease

Scott M. Bartell, John Longhurst, Thomas Tjoa, Constantinos Sioutas, Ralph J. Delfino

TABLE OF CONTENTS

	Page
Mean-centered exposure explanation	2
Table S1: Spearman correlation matrix for outdoor home air pollutant exposures	4
Table S2: Associations of supraventricular tachycardia with outdoor air pollutants	5
Table S3: Associations of hourly heart rate variability with outdoor air pollutants	7

Mean centered exposure explanation

We used mean centered exposures to adjust for between-subject and between-phase exposure effects as proposed by Sheppard et al. (2005). The focus of the analysis is on the effect of within-subject, within-phase mean centered exposure. This approach is summarized here.

Group outdoor air pollutant exposures are assigned to each participant in each of their two phases of study (5-day ambulatory monitoring periods). Pollutant associations with HRV or arrhythmia measurements can be decomposed into three separate components: the between-subject effect; the within-subject, between-phase effect; and the within-subject, within-phase effect. The between-subject effect of exposure is the degree to which an outcome is associated with differences in the air pollutants across participants. This effect estimate is potentially confounded by time-independent factors, such as the region of the retirement community or health-related activities in the community or among some participants. The within-subject, between-phase effect of exposures effect is the degree to which an outcome is associated with differences in the air pollutants across phases. Because the study phases occurred at different times for each subject, this effect estimate may be confounded by season. The within-phase, within-subject effect of exposure is the degree to which an outcome is associated with differences in the air pollutants when comparing across daily (or hourly) measurements within the same 5-day phase for the same participant. This third component of the exposure-outcome relationship is less susceptible to confounding by unmeasured factors that differ by season or by participant.

The following variables are entered as predictors in each model in order to accommodate the three components of the exposure-outcome relationship:

$\overline{X}_k$, the average exposure for subject *k*. The coefficient for this variable is an estimate of the between-subject component of the exposure-outcome relationship.

$\overline{X}_{jk} - \overline{X}_k$, the average exposure for subject *k* in phase *j* minus the average exposure for subject *k*. The coefficient for this variable is an estimate of the within-subject, between-phase component of the exposure-outcome relationship.

$X_{jkm} - \overline{X}_{jk}$, the current or lagged exposure corresponding to the outcome measurement on day (or hour) *m* for subject *k* in phase *j* minus the average exposure for subject *k* in phase *j*. The coefficient for this variable is an estimate of the within-subject, within-phase component of the exposure-response relationship. All of our exposure-response estimates reflect this third component.

Reference: Sheppard L, Slaughter JC, Schildcrout J, Liub LJS, Lumley T. 2005. Exposure and measurement contributions to estimates of acute air pollution effects. J Expos Anal Environ Epidemiol 15: 366-376.

Supplemental Material, Table S1. Spearman correlation matrix for outdoor home air pollutant exposures.[a]

	$PM_{2.5}$	Organic carbon	Black carbon	Primary OC	Secondary OC	$PM_{0.25}$	$PM_{0.25-2.5}$	$PM_{2.5-10}$	NO_x	CO	O_3
Particle number	-0.13	0.27	0.40	0.47	-0.08	0.36	-0.12	0.06	0.63	0.45	-0.38
$PM_{2.5}$	1.00	0.44	0.58	0.43	0.22	0.20	0.87	0.55	0.14	0.31	0.04
Organic carbon		1.00	0.63	0.65	0.72	0.41	0.33	0.33	0.46	0.59	-0.05
Black carbon			1.00	0.88	0.07	0.52	0.43	0.44	0.83	0.79	-0.38
Primary OC				1.00	0.01	0.55	0.33	0.36	0.79	0.75	-0.36
Secondary OC					1.00	0.09	0.16	0.15	-0.09	0.11	0.26
$PM_{0.25}$						1.00	0.17	0.35	0.51	0.54	0.01
$PM_{0.25-2.5}$							1.00	0.60	0.01	0.13	0.08
$PM_{2.5-10}$								1.00	0.18	0.26	0.06
NO_x									1.00	0.82	-0.53
CO										1.00	-0.29

[a] All exposures are 24-hr averages and are mean-centered by retirement community and seasonal phase.

Supplemental Material, Table S2. Associations[a] of supraventricular tachycardia with outdoor air pollutants, per IQR

Exposure and averaging time	Daily RR (95% CI)	Hourly OR (95% CI)
Particle number		
1-hr	--	1.02 (0.79, 1.31)
4-hr	--	1.00 (0.74, 1.34)
8-hr	--	1.02 (0.74, 1.40)
24-hr	1.14 (0.78, 1.66)	0.96 (0.49, 1.87)
3-day	2.07 (0.50, 8.55)	1.52 (0.42, 5.49)
5-day	0.41 (0.08, 2.22)	0.83 (0.05, 12.76)
Black carbon		
1-hr	--	0.86 (0.72, 1.02)*
4-hr	--	0.86 (0.73, 1.02)*
8-hr	--	0.92 (0.77, 1.11)
24-hr	0.92 (0.74, 1.13)	0.93 (0.66, 1.32)
3-day	1.15 (0.72, 1.85)	0.83 (0.46, 1.48)
5-day	0.95 (0.42, 2.16)	0.99 (0.49, 1.99)
Elemental carbon		
1-hr	--	0.98 (0.88, 1.09)
4-hr	--	0.89 (0.76, 1.04)
8-hr	--	0.90 (0.74, 1.09)
24-hr	0.98 (0.80, 1.21)	0.97 (0.75, 1.25)
3-day	1.39 (0.95, 2.03)*	0.93 (0.50, 1.75)
5-day	1.12 (0.54, 2.32)	1.25 (0.55, 2.84)
Organic Carbon (OC)		
1-hr	--	0.94 (0.62, 1.41)
4-hr	--	0.90 (0.54, 1.51)
8-hr	--	1.00 (0.53, 1.89)
24-hr	0.87 (0.54, 1.42)	0.76 (0.32, 1.82)
3-day	0.83 (0.32, 2.16)	0.51 (0.15, 1.73)
5-day	0.32 (0.04, 2.60)	0.29 (0.07, 1.29)
Primary OC		
1-hr	--	0.93 (0.71, 1.22)
4-hr	--	0.89 (0.67, 1.20)
8-hr	--	0.87 (0.49, 1.55)
24-hr	1.18 (0.58, 2.40)	0.95 (0.30, 3.02)
3-day	3.57 (0.98, 13.01)*	1.53 (0.31, 7.62)
5-day	8.11 (1.16, 56.66)**	1.22 (0.22, 6.82)
Secondary OC		
1-hr	--	0.96 (0.75, 1.23)
4-hr	--	1.06 (0.75, 1.48)
8-hr	--	1.12 (0.77, 1.62)
24-hr	0.83 (0.57, 1.21)	0.81 (0.37, 1.76)
3-day	0.48 (0.24, 0.94)**	0.65 (0.20, 2.13)
5-day	0.58 (0.29, 1.16)	0.64 (0.31, 1.35)
$PM_{2.5}$		
1-hr	--	1.01 (0.76, 1.35)
4-hr	--	1.02 (0.75, 1.40)
8-hr	--	0.99 (0.74, 1.34)

Exposure and averaging time	Daily RR (95% CI)	Hourly OR (95% CI)
24-hr	0.77 (0.54, 1.09)	0.85 (0.62, 1.16)
3-day	0.80 (0.40, 1.61)	0.66 (0.36, 1.18)
5-day	0.87 (0.39, 1.94)	0.45 (0.18, 1.14)*
$PM_{0.25}$		
24-hr	0.94 (0.76, 1.17)	--
Lag 25-48 hr	0.99 (0.79, 1.24)	--
2-day	0.90 (0.67, 1.21)	--
$PM_{0.25-2.5}$		
24-hr	0.89 (0.66, 1.21)	--
Lag 25-48 hr	1.20 (0.98, 1.47)*	--
2-day	1.21 (0.87, 1.68)	--
$PM_{2.5-10}$		
24-hr	0.88 (0.71, 1.10)	--
Lag 25-48 hr	0.97 (0.80, 1.18)	--
2-day	0.91 (0.66, 1.24)	--
O_3		
1-hr	--	1.16 (0.99, 1.37)*
4-hr	--	1.19 (1.02, 1.39)**
8-hr	--	1.25 (1.00, 1.56)**
24-hr	1.15 (0.80, 1.64)	0.89 (0.50, 1.57)
3-day	0.46 (0.22, 0.95)**	0.33 (0.10, 1.17)*
5-day	0.77 (0.23, 2.64)	0.31 (0.04, 2.54)
NO_x		
1-hr	--	0.82 (0.65, 1.02)*
4-hr	--	0.77 (0.63, 0.94)**
8-hr	--	0.92 (0.74, 1.15)
24-hr	1.05 (0.81, 1.35)	1.22 (0.74, 2.01)
3-day	1.76 (1.00, 3.13)*	1.51 (0.60, 3.80)
5-day	0.94 (0.37, 2.40)	2.51 (0.55, 11.54)
CO		
1-hr	--	0.75 (0.60, 0.93)[#]
4-hr	--	0.64 (0.48, 0.87)[#]
8-hr	--	0.79 (0.60, 1.05)*
24-hr	0.92 (0.68, 1.23)	0.89 (0.57, 1.40)
3-day	1.54 (0.92, 2.59)	0.90 (0.41, 1.97)
5-day	1.72 (0.59, 4.98)	1.22 (0.45, 3.29)

[a] All models are adjusted for adjusted for average actigraph-derived physical activity and heart rate, temperature of the same lag average, day of week, seasonal study phase (mean centered exposure), and community group (mean centered exposure), using generalized estimating equations. Daily RRs use Poisson log-link models with daily VT counts as the outcome, and hourly ORs use binomial logit-link models with hourly absence/presence of any VT as the outcome.

* $p < 0.1$, ** $p < 0.05$, [#] $p < 0.01$

Supplemental Material, Table S3. Associations[a] of hourly heart rate variability with outdoor air pollutants.

Exposure Averaging Time	SDNN Coefficient (95% CI)[a]	rMSSD Coefficient (95% CI)	Logit pNN50 Coefficient (95% CI)
Particle number			
1-hr	-0.51 (-1.46, 0.44)	-0.03 (-0.85, 0.80)	-0.010 (-0.052, 0.032)
4-hr	-0.40 (-1.71, 0.92)	-0.07 (-1.57, 1.43)	-0.024 (-0.094, 0.047)
8-hr	-1.29 (-2.97, 0.38)	0.55 (-1.69, 2.79)	-0.017 (-0.118, 0.084)
24-hr	-0.92 (-3.79, 1.95)	-0.26 (-4.74, 4.22)	0.011 (-0.182, 0.204)
3-day	1.70 (-4.18, 7.57)	-0.71 (-9.74, 8.32)	0.224 (-0.142, 0.590)
5-day	9.26 (1.23, 17.30)**	2.03 (-10.12, 14.17)	0.192 (-0.347, 0.730)
Black carbon			
1-hr	0.40 (-0.28, 1.07)	0.03 (-0.59, 0.66)	0.028 (-0.004, 0.060)*
4-hr	0.60 (-0.25, 1.46)	0.55 (-0.51, 1.61)	0.048 (-0.003, 0.100)*
8-hr	0.55 (-0.45, 1.55)	1.20 (-0.20, 2.60)*	0.051 (-0.014, 0.116)
24-hr	0.29 (-1.11, 1.69)	0.03 (-2.16, 2.22)	0.053 (-0.046, 0.153)
3-day	0.45 (-2.03, 2.94)	1.05 (-2.86, 4.97)	0.133 (-0.045, 0.311)
5-day	0.03 (-4.20, 4.26)	5.51 (-1.17, 12.19)	0.159 (-0.132, 0.450)
Elemental carbon			
1-hr	0.22 (-0.46, 0.91)	0.29 (-0.31, 0.88)	0.029 (-0.003, 0.061)*
4-hr	0.94 (0.02, 1.86)**	0.42 (-0.69, 1.53)	0.044 (-0.012, 0.099)
8-hr	0.78 (-0.30, 1.85)	0.83 (-0.66, 2.33)	0.073 (0.002, 0.143)**
24-hr	0.31 (-1.16, 1.78)	-0.06 (-2.39, 2.27)	0.067 (-0.042, 0.175)
3-day	-0.011 (-2.53, 2.51)	0.82 (-3.00, 4.64)	0.120 (-0.058, 0.299)
5-day	-3.15 (-7.27, 0.97)	3.67 (-2.19, 9.53)	0.203 (-0.074, 0.480)
Organic Carbon (OC)			
1-hr	1.16 (-0.72, 3.03)	0.55 (-1.08, 2.19)	0.061 (-0.027, 0.150)
4-hr	1.34 (-1.15, 3.84)	0.37 (-2.73, 3.47)	0.081 (-0.075, 0.238)
8-hr	1.16 (-1.68, 4.00)	-0.82 (-4.85, 3.20)	0.075 (-0.120, 0.270)
24-hr	0.68 (-2.64, 3.99)	1.21 (-3.97, 6.39)	0.207 (-0.043, 0.457)
3-day	-1.39 (-6.11, 3.32)	0.28 (-6.93, 7.50)	0.192 (-0.155, 0.540)
5-day	-5.75 (-13.09, 1.58)	-0.39 (-10.88, 10.09)	-0.032 (-0.529, 0.466)
Primary OC			
1-hr	0.94 (-1.06, 2.94)	0.73 (-1.02, 2.49)	0.070 (-0.024, 0.163)
4-hr	2.62 (-0.06, 5.31)*	1.40 (-1.82, 4.62)	0.036 (-0.124, 0.196)
8-hr	2.06 (-1.12, 5.25)	3.23 (-1.16, 7.62)	0.111 (-0.098, 0.321)
24-hr	-0.21 (-4.98, 4.57)	0.06 (-7.44, 7.57)	0.241 (-0.111, 0.592)
3-day	-0.90 (-8.33, 6.53)	3.78 (-7.28, 14.84)	0.388 (-0.131, 0.906)
5-day	-13.1 (-24.15, -2.11)**	0.64 (-14.33, 15.62)	0.430 (-0.294, 1.154)
Secondary OC			
1-hr	-0.06 (-1.14, 1.03)	-0.06 (-0.90, 0.78)	-0.001 (-0.047, 0.044)
4-hr	-1.44 (-3.05, 0.17)*	-0.74 (-2.56, 1.07)	-0.033 (-0.126, 0.060)
8-hr	-1.16 (-3.11, 0.79)	-3.10 (-5.62, -0.58)**	-0.133 (-0.257, -0.009)**
24-hr	0.29 (-2.35, 2.93)	1.46 (-2.44, 5.35)	0.076 (-0.112, 0.265)
3-day	-2.17 (-5.94, 1.59)	-0.88 (-6.50, 4.73)	-0.069 (-0.334, 0.196)
5-day	-1.53 (-6.09, 3.03)	-1.61 (-8.07, 4.86)	-0.139 (-0.443, 0.165)
$PM_{2.5}$			
1-hr	0.28 (-0.65, 1.22)	0.04 (-0.98, 1.06)	0.023 (-0.028, 0.075)
4-hr	0.78 (-0.28, 1.85)	0.73 (-0.74, 2.20)	0.013 (-0.056, 0.082)

Exposure Averaging Time	SDNN Coefficient (95% CI)[a]	rMSSD Coefficient (95% CI)	Logit pNN50 Coefficient (95% CI)
8-hr	0.33 (-0.84, 1.49)	-0.01 (-1.76, 1.74)	-0.038 (-0.118, 0.041)
24-hr	0.076 (-1.34, 1.49)	-1.23 (-3.48, 1.02)	0.007 (-0.093, 0.107)
3-day	-0.31 (-2.29, 1.66)	-1.04 (-4.25, 2.17)	0.004 (-0.134, 0.143)
5-day	-2.38 (-5.85, 1.09)	0.11 (-5.37, 5.58)	-0.024 (-0.267, 0.219)
O_3			
1-hr	-0.19 (-1.12, 0.74)	-0.83 (-1.84, 0.17)	-0.046 (-0.098, 0.006)*
4-hr	-0.22 (-1.27, 0.84)	-0.84 (-2.17, 0.49)	-0.019 (-0.084, 0.046)
8-hr	-0.084 (-1.36, 1.20)	-0.59 (-2.33, 1.15)	-0.006 (-0.089, 0.077)
24-hr	2.42 (-0.32, 5.16)*	0.63 (-3.53, 4.79)	0.135 (-0.056, 0.325)
3-day	-0.047 (-4.54, 4.45)	-1.95 (-8.94, 5.04)	-0.076 (-0.392, 0.240)
5-day	-10.68 (-18.32, -3.05)#	-10.48 (-22.31, 1.34)*	-0.490 (-1.029, 0.049)*
NO_x			
1-hr	0.29 (-0.64, 1.22)	0.68 (-0.20, 1.55)	0.035 (-0.009, 0.078)
4-hr	0.53 (-0.62, 1.68)	1.25 (-0.10, 2.60)*	0.072 (0.009, 0.136)**
8-hr	1.13 (-0.24, 2.50)	2.60 (0.76, 4.43)#	0.113 (0.030, 0.196)#
24-hr	0.67 (-1.58, 2.91)	0.81 (-2.62, 4.24)	0.102 (-0.049, 0.253)
3-day	2.36 (-1.89, 6.61)	3.68 (-2.91, 10.27)	0.305 (0.011, 0.599)**
5-day	5.42 (-1.40, 12.25)	10.19 (-0.50, 20.89)*	0.383 (-0.082, 0.848)
CO			
1-hr	-0.30 (-1.30, 0.70)	0.94 (-0.00, 1.89)*	0.043 (-0.005, 0.091)*
4-hr	0.18 (-1.03, 1.40)	1.62 (0.22, 3.03)**	0.071 (0.003, 0.139)**
8-hr	0.85 (-0.59, 2.29)	2.37 (0.46, 4.27)**	0.101 (0.013, 0.190)**
24-hr	-0.29 (-2.38, 1.80)	0.02 (-3.06, 3.11)	0.097 (-0.044, 0.239)
3-day	0.45 (-2.88, 3.79)	1.59 (-3.42, 6.61)	0.134 (-0.100, 0.367)
5-day	-0.71 (-6.18, 4.75)	2.45 (-5.79, 10.68)	0.092 (-0.282, 0.466)

[a] Hourly ECG HRV outcomes in relation to air pollutants also measured on an hourly basis. Pollutant exposures are moving averages for the hour including the HRV measurement as well as previous hours for exposures with averaging times of more than one hour. All models are adjusted for adjusted for average actigraph-derived physical activity and heart rate for the same hour, temperature of the same lag average, day of week, time of day, seasonal study phase (mean centered exposure), and community group (mean centered exposure).

* $p < 0.1$, ** $p < 0.05$, # $p < 0.01$
